# Frequency and association of Epstein-Barr Virus genotype in rheumatoid arthritis patients of Khyber Pakhtunkhwa, Pakistan

**DOI:** 10.1371/journal.pone.0295124

**Published:** 2023-12-20

**Authors:** Ayesha Munir, Suleman Khan, Sanaullah Khan, Sobia Attaullah, Mehwish Munir, Aisha Saleem, Ijaz Ali

**Affiliations:** 1 Department of Zoology, University of Peshawar, Peshawar, Khyber Pakhtunkhwa, Pakistan; 2 Department of Rheumatology, Lady Reading Hospital, Peshawar, Khyber Pakhtunkhwa, Pakistan; 3 Islamia College (University) Peshawar, Peshawar, Khyber Pakhtunkhwa, Pakistan; 4 Abdul Wali Khan University Mardan, Mardan, Khyber Pakhtunkhwa, Pakistan; 5 Department of Biosciences, COMSATS University Islamabad, Islamabad, Pakistan; 6 CAMB, College of Arts and Sciences, Gulf University for Science and Technology, Hawally, Kuwait; Mie University Hospital: Mie Daigaku Igakubu Fuzoku Byoin, JAPAN

## Abstract

**Background:**

Rheumatoid arthritis (RA) is an immune-mediated, polyarthritis linked with various genetic and environmental causative agents. Among environmental triggers, Epstein-Barr Virus (EBV) is considered the most potent etiological agent.

**Objective:**

This study aimed to investigate the prevalence of EBV and its genotypes in RA patients and to investigate their association with clinical and laboratory parameters of RA.

**Methodology:**

This study included blood samples of RA and control healthy individuals (100 each). Blood samples along with clinical and laboratory parameters were collected from patients after consent in the Department of Rheumatology, at Lady Reading Hospital, in Peshawar Pakistan. Blood samples were processed for DNA extraction followed by PCR amplification for EBV detection and genotype discrimination.

**Results:**

RA patients were 85 females and 15 males with a mean age of 40.13±14.05 years. EBV Type-1 was detected in 45% of RA and 9% of control cases. The mean disease duration of RA patients was 6.61±6.23 years. Out of 100 diseased patients, 43% were seropositive rheumatoid arthritis (SPRA) and showed a significant correlation with a family history of RA in EBV-positive individuals (P = 0.017). The demographic, clinical, and laboratory parameters of RA patients showed a non-significant association with EBV. Moreover, only a family history and Serum creatinine of RA patients showed a significant association with EBV (P = 0.0001 and P = 0.022 respectively).

**Conclusion:**

It is concluded that EBV-1 is prevalent and associated with RA. Further investigation is required for detailed genetic analysis of EBV to determine its possible role in modulating the immune system in RA.

## Introduction

Rheumatoid arthritis (RA) is a chronic autoimmune inflammatory disorder, that predominantly impairs synovial joints affecting 0.5–1% of the world’s adult population with women being highly prone to be suffered [1]. RA is categorized into two major subsets: seropositive rheumatoid arthritis (SPRA) and seronegative rheumatoid arthritis (SNRA) characterized by the presence or absence of autoantibodies [2]. It is presumed that RA has been linked with several genetic and environmental factors including infectious agents with EBV considered the utmost potent agent in the onset of RA [3]. EBV is a DNA-containing ubiquitous Herpes virus affecting 90% of the world’s adult population [4]. It has been classified into two genotypes, type 1 and type 2 based on the EBNA-2 gene which reveals only 54% similarity [5]. EBV latently persists in the body for a lifetime expressing genes involved in mononucleosis and various EBV-associated carcinomas such as Burkitt lymphoma and Hodgkin lymphoma etc [6].

Several potential mechanisms have been proposed to elucidate the immunomodulatory actions of EBV in the arthritogenicity of RA [7]. Although, it has been suggested that molecular mimicry of EBV antigenic envelop and nuclear amino acid sequence with human leukocyte antigen (HLA) and synovial protein sequence respectively, could be a potential trigger for autoimmunity in a genetically predisposed RA individual [8]. Moreover, EBV- infected RA patients have an aberrant cytotoxic T-cell response and high titer of antibodies to EBV antigens i.e. Epstein-Barr nuclear antigen (EBNA), viral capsid antigen, and early antigen [9].

Several studies speculated that EBV infection is strongly associated with RA from different parts of the world [7, 10, 11]. In Pakistan, EBV has been detected in various lymphoma patients from different areas [12–14]. It is reported that RA is prevalent and increasing at a rate of 5.5% per year with a prevalence rate of 12.9–26.9% [15]. No data regarding EBV prevalence in RA patients in Pakistan is available to date, therefore this study is designed to find the frequency of EBV and its genotypes in the blood of RA patients and to investigate its possible association with RA clinical and laboratory parameters.

## Material and methods

### Study population

This cross-sectional study comprised of 100 confirmed RA patients and 100 healthy individuals with no RA signs and symptoms. Confirmed RA samples diagnosed by a rheumatologist with established classification criteria of 2010 American College of Rheumatology/European League Against Rheumatism [16] were recruited from the Department of Rheumatology, Lady Reading Hospital Peshawar Khyber Pakhtunkhwa Pakistan between 21 July 2022, and 19 August 2023. The study protocol was approved by the Ethics Committee of Department of Pharmacy, Faculty of Life and Environmental Sciences, University of Peshawar (approval numbers, 520/EC-FLES-UOP/2022), and all patients gave consent for the use of their blood in the present study.

### Data and sample collection

Clinical and laboratory parameters including C-reactive protein (CRP), white blood cells (WBC), red blood cells (RBC), platelets (PLT), Alanine transaminase (ALT), Alkaline phosphatase (ALP), serum creatinine, urea and type of RA (SPRA or SNRA) were retrieved from the patient’s clinical files while primary data including disease duration, family history, and type of immunosuppressive medications, etc. was collected through a predesigned questionnaire after prior consent from each patient. Peripheral blood (~2.5 mL) was collected from the study population under sterile conditions in EDTA blood tubes and was transferred to the Laboratory of Virology and Immunology, Department of Zoology, University of Peshawar Pakistan for EBV detection and genotyping.

### DNA extraction and detection

The EBV DNA was isolated using GeneJET Genomic DNA Purification Kit (Thermo Scientific™, USA) according to the manufacturer’s instructions. The DNA was stored at -70°C until further process. The extracted DNA was initially subjected to PCR for amplification using primers PCO3/PCO4 (b-globulin) to confirm the extracted DNA quality (internal control). After that nested PCR was carried out for discrimination of EBV genotypes (type 1 and type 2) using specific primers of EBNA-3C following the protocol of Sharifpour et al. [17]. The first round of PCR was executed in a 25μL mixture, comprising of 10μL of extracted DNA, 2.5μL PCR buffer (10X), 0.5μL dNTPs (10mM), 3μL MgCl_2_ (25mM), 6.8μL molecular grade dH_2_O, 0.2μL *Taq* DNA Polymerase (5U/μL) (Fermentas USA) and 1μL (10pmol) of each forward and reverse primer and the reaction was run in a thermal-cycler (Bio-Rad, USA). The condition of nested round was same except the primers and cDNA of first round (4μL) was used. The PCR program of 1^st^ and nested round were same as 95°C for 10 min followed by 35 cycle of 94°C for 45 s, 54.5°C for 45 s and 72°C for 45 s with final extension at 72°C for 10 min.

The nested round PCR product was separated on 1.5% agarose gel and viewed under UV light using a trans-illuminator. The nested PCR product (75bp for EBV type 1 and 168 for EBV type 2) was compared with the DNA ladder (50bp) (**S1 Fig**).

### Statistical analysis

The obtained results were analyzed using statistical packages for social sciences version 25 (SPSS, Inc, Chicago, IL, USA). Data was analyzed using the Chi-square test for gender, family history and immunosuppressive drugs parameters while independent *t-*test was applied on continuous variables such as age, and clinical parameters etc. P value less than 0.05 was considered significant.

## Results

Two hundred individuals were enrolled in this study; 100 RA including 15 males and 85 females with a mean age of 40.13±14.05 years and 100 randomly selected age-matched control individuals including 44 males and 56 females under a mean age of 42.44±12.21. The mean disease duration of RA patients was 6.61±6.23 years. Overall, 45% of RA samples and 9% of control cases were found positive for EBV DNA by nested PCR with significant association (P = 0.00001). All EBV-positive RA patients were confirmed having EBV-1 genotypes (**Table 1**).

**Table 1:** Demographics and laboratory parameters of RA patients.

Parameter	Frequency	
**Sex (Male/Female)**	15/85	
**SPRA/SNRA**	86/14	
**RA family history (positive/ negative)**	21/79	
**Variables**	**Mean (±Sd)**	**Range**
**Age (years)**	40.13 ± 14.05	18–83
**Disease duration (years)**	6.61 ± 6.23	0.5–30
**CRP (mg/L)**	8.7 ± 6.87	0.3–48.2
**WBC (×10** ^**3**^ **/ uL)**	9.8 ± 3.19	3.2–26.2
**RBC (×10** ^**6**^ **/ uL)**	4.6 ± 0.81	3.2–10
**PLT count (×10** ^**3**^ **/ uL)**	398.8 ± 143.1	118–805
**ALT (U/L)**	24.32 ± 12.49	3–85
**ALP (U/L)**	108.8 ± 51.4	29–409
**Serum creatinine (mg/dL)**	0.69 ± 0.20	0.24–1.3
**Urea (mg/dL)**	28.8 ±10.3	10–46

**Fig 1:**
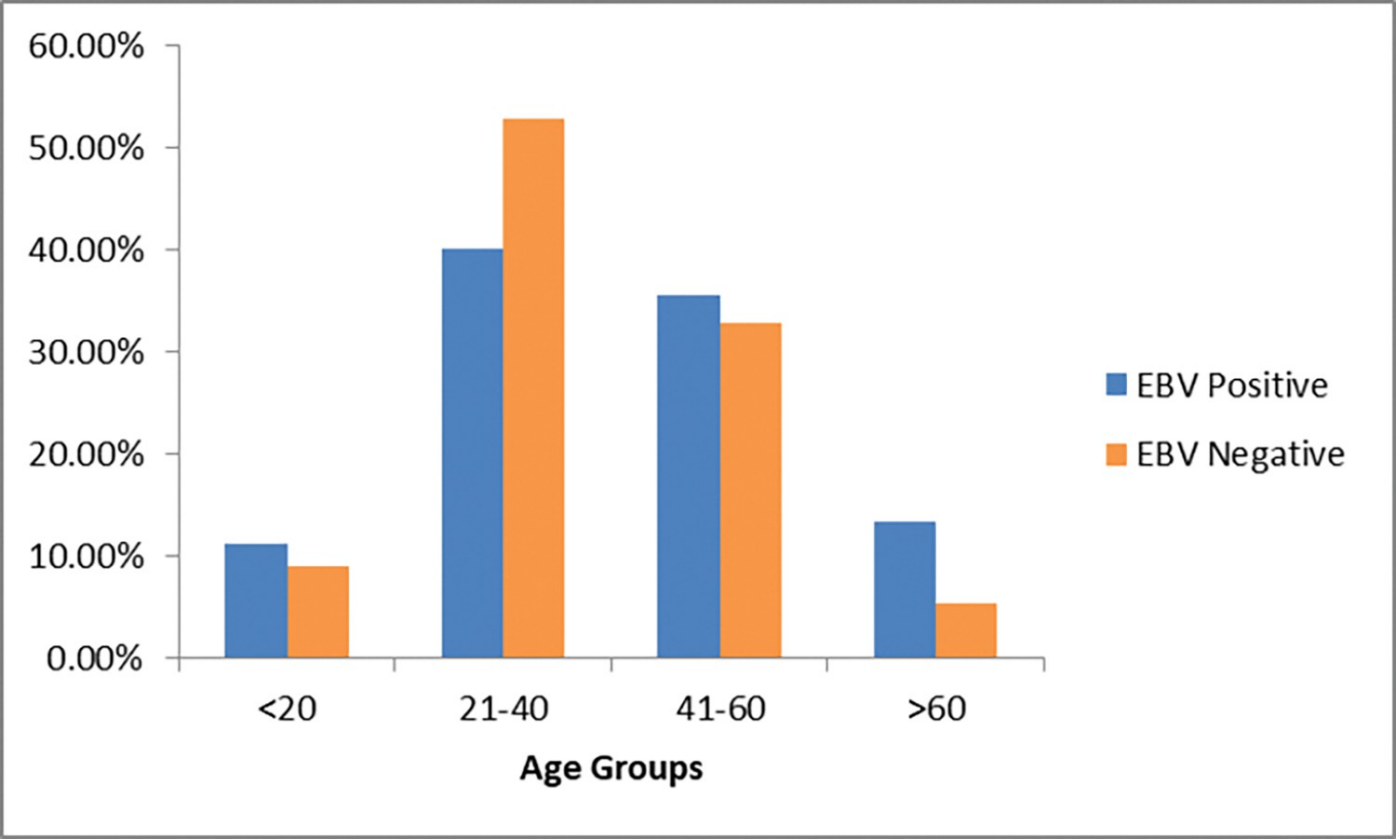
Prevalence of EBV in different age groups of RA patients.

EBV positivity percentage in each age group of RA is shown in **Fig 1**. The Prevalence of EBV was higher in females (82%) than in males (18%) RA patients (P = 0.481). The majority of RA patients were under the age range 21–40 years followed by the 41–60 years’ age group. EBV detection in the age group ≥60 years was comparatively higher than in the other groups. No statistically significant association was observed between age and EBV presence (P = 0.343).

EBV positive and negative RA patients showed no significant difference concerning clinical and laboratory parameters such as CRP (P = 0.313), WBC (P = 0.249), RBC (P = 0.171), PLT (P = 0.729), ALT (P = 0.200), ALP (P = 0.067), and urea (P = 0.046) except serum creatinine (P = 0.022), shown in Table 2.

**Table 2:** Clinical and demographic characteristics of RA patients in correlation with EBV.

Parameters	EBV positive	EBV negative	p- value
	n = 45	n = 55	
**Gender (female/male)**	82/18 (%)	87/13 (%)	0.481*
**Age (years)**	41.55 ± 14.83	38.89 ± 13.09	0.343
**CRP (mg/L)**	8.02 ± 2.11	9.26 ± 7.98	0.313
**WBC (×103/ μL)**	10.24 ± 3.69	9.50 ± 2.67	0.249
**RBC (×106/ μL)**	4.73 ± 1.07	4.51 ± 0.46	0.171
**PLT count (×103/ μL)**	404.37 ± 146.92	394.38 ± 139.85	0.729
**ALT (U/L)**	26.08 ± 12.04	22.87 ± 12.66	0.200
**ALP (U/L)**	98.46 ± 29.94	117.30 ± 62.58	0.067
**Serum creatinine (mg/dL)**	0.64 ± 0.17	0.73 ± 0.21	0.022
**Urea (mg/dL)**	26.55 ± 10.58	30.61 ± 9.56	0.046

*Chi square test

Patients were further characterized based on the drug of choice; the number of patients using Disease-modifying anti-rheumatic drugs (DMARDs) was comparatively higher than patients using biologics and steroid-DMARDs category. EBV prevalence was found higher in biologics-taking patients (60%) compared to DMARDs (43%) and steroid-DMARDs category (42.3%) (P = 0.599). Among 100 RA samples, 21 had a positive family history of RA within which 81% were positive for EBV showing a significant (P = 0.0001) correlation with RA (**Table 3**). Out of 86 SPRA patients, 43% were positive for EBV. Moreover, a significant association of the RA major subset (SPRA and SNRA) was found with a family history of RA in EBV-positive RA individuals (P = 0.017) (**Table 4**).

**Table 3:** Association of EBV with RA immunosuppressive type of treatment, family history and type of RA (SPRA and SNRA).

Immunosuppressive drug categories (n)	EBV positive	EBV negative	p- value
	n(%)	n(%)	
DMARDs (64)	28 (43.8)	36 (56.2)	0.599
DMARDs-steroids (26)	11 (42.3)	15 (57.7)	
Biologics (10)	6 (60)	4 (40)	
**Family history (n)**			**<0.001**
Positive (21)	17 (81)	4 (19)	
Negative (79)	28 (35)	51 (64)	

**Table 4:** Association of EBV, family history and type of RA (SPRA and SNRA).

Parameters		Presence of autoantibodies (RA and Anti- CCP)		Total	p- value
		SPRA (n = 86)	SNRA (n = 14)		
**EBV Positive**	Positive RA family history	11	6	17	**0.017**
		64.7%	35.3%		
	Negative RA family history	26	2	28	
		92.9%	7.1%		
Total		37	8	45	
		82.2%	17.8%		
**EBV Negative**	Positive RA family history	3	1	4	0.348
		75.0%	25.0%		
	Negative RA family history	46	5	51	
		90.2%	9.8%		
Total		49	6	55	
		89.1%	10.9%		

## Discussion and conclusion

RA patients showed strong susceptibility to a variety of diseases that either worsen their overall complications or contribute to their pathogenesis causing arthralgia and arthritis [18]. This may lead to complications added by poor health and hygienic conditions contributing to various viruses causing such diseases [19]. The viruses associated with RA may spread through unscreened blood transfusion, saliva, sharing of drinks/eating utensils, and other personal use items [20]. In the current study, the prevalence of EBV was 45% in RA and 9% in control individuals. Likewise, a 79% EBV prevalence rate was detected in the Sardinian RA population conducted by Erre et al. [6]. In line with our findings, other researchers found 40% [21] and 29.2% [22] EBV prevalence rate in the RA population. The variation in findings of mentioned studies is partly attributable to the differences in the prevalence rate of EBV and RA worldwide.

RA patients’ samples were further analyzed for EBV genotype specification. According to our findings, only the type 1 genotype of EBV was prevalent in RA patients. A study performed in Pakistan on lymphoma patients detected both genotypes, with type 1 being most prevalent (90%) than type 2 (9%) [12]. Similarly, another study [23] observed 60% of type 1 and 26% of type 2 EBV genotypes in transgender sex workers in Pakistan. Likewise, Salahuddin et al. [24] also reported a high prevalence of the type- 1 (40%) genotype compared to type- 2 (15%) in HIV-infected Pakistani transsexual individuals. To our knowledge, this is the first time to perform detection of the EBV genotype in RA patients. One of the possible explanations for the 100%, type 1 genotype in our study is due to the high prevalence of only type 1 genotype in our area.

In this study, no significant relationship has been observed between RA demographic, clinical, and laboratory parameters such as gender, age, RA family history, disease duration, CRP, WBC, RBC, PLT, ALT, ALP, Urea, Type of immunosuppressive therapy, family history and type of RA (SPRA and SPNA) with EBV presence except serum creatinine. The study carried out by Costenbader and Karlson [25] also demonstrated that the presence of EBV is not influenced by the presence or absence of RF, age, duration of RA, disease activity, or RA treatment. In agreement, Hamid [26] did not report any correlation between the seroprevalence of EBV and some RA parameters i.e., CRP, ESR, and WBCs count in blood, which might infer that EBV plays a role in the onset of RA, but have no link with further steps in the pathogenesis of RA.

Therapies for the treatment of RA are very effective but can initiate multiple mechanisms affecting immune surveillance of latently EBV-infected cells initiating continuous viral reactivation and persistent immune stimulation mostly in long-term treatment [27]. Our study showed no significant correlation of drug category with the presence of EBV while biologics using RA patients tended to have comparatively high EBV seropositivity but this did not reach significance. Correspondingly, a study with similar findings compared the seropositivity of EBV in patients taking immunosuppressive drugs such as steroids and DMARDs observing no significant association [28]. In accordance, other research studies [7, 29, 30] also didn’t show any significant positive correlation between EBV and RA medication including biologics and DMARDs.

Moreover, this work indicates the significant correlation of EBV presence with family history and major subtypes of RA (SPRA and SNRA). However, there was no supporting evidence found from other studies regarding the link of EBV with RA family history and its combined association with RA subtypes. This finding could be explained by the possibility that the inheritance of susceptible genes involved in the onset of RA may contribute to molecular mimicry between EBV and self-proteins leading to a loss of immune tolerance in the predisposed host [31].

In summary, this study showed that RA patients have a high prevalence of EBV and especially the type 1 genotype thus suggesting EBV’s significant role as a trigger in the onset or exacerbation of RA. Additional efforts are required for genetic analysis of RA-associated genes in patients carrying EBV as an agent for acquiring RA. This research further suggests the genetic analysis of EBV i.e. different strains and variant investigation present in our area involved in RA as well as the study on preventive strategies for interrupting EBV transmissions such as vaccine and other antiviral drug development that would be effective for halting RA.

## Supporting information

S1 Fig (TIF)

S1 Raw image (PDF)
