# Supplementary figures and images for: Frequency and association of Epstein-Barr Virus genotype in rheumatoid arthritis patients of Khyber Pakhtunkhwa, Pakistan
Source: PLoS One. 2023 Dec 20;18(12):e0295124. doi: 10.1371/journal.pone.0295124 (PMC10732363; doi:10.1371/journal.pone.0295124)

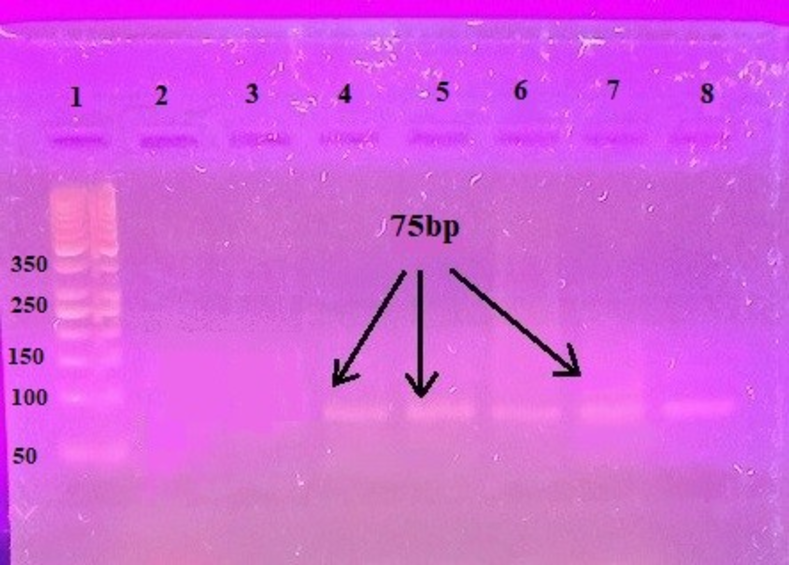

Supplement: S1 Fig — (TIF) [file pone.0295124.s001.tif]

**A B C D E F G H**

**50 bp**

**I**

**J**

**K**

**50 bp**

**A-G, I, J: 75 bp**

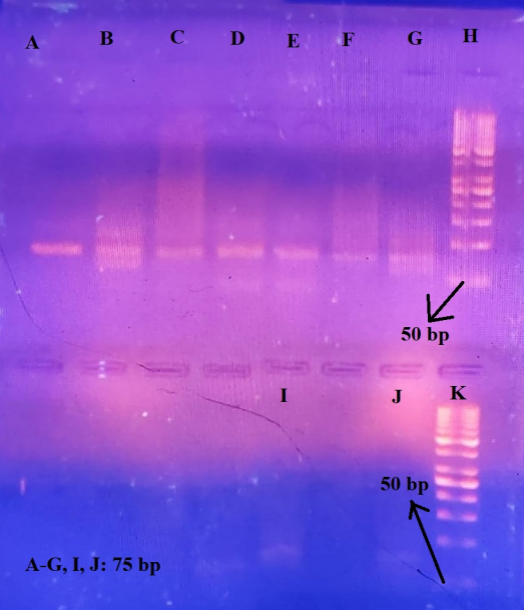

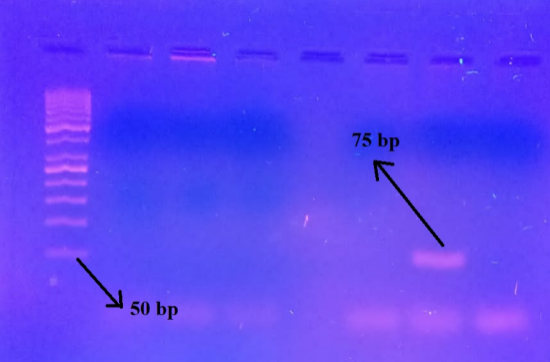

75 bp

50 bp

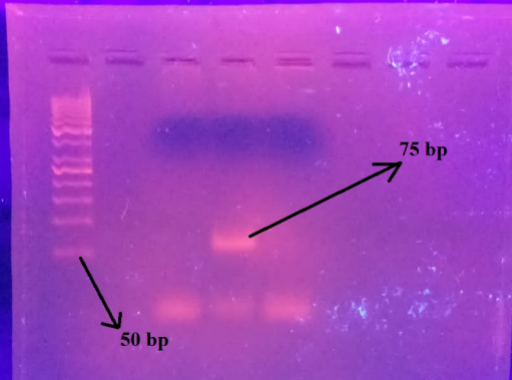

1 2 3 4 5 6

50 bp

1-3, 6: 75 bp

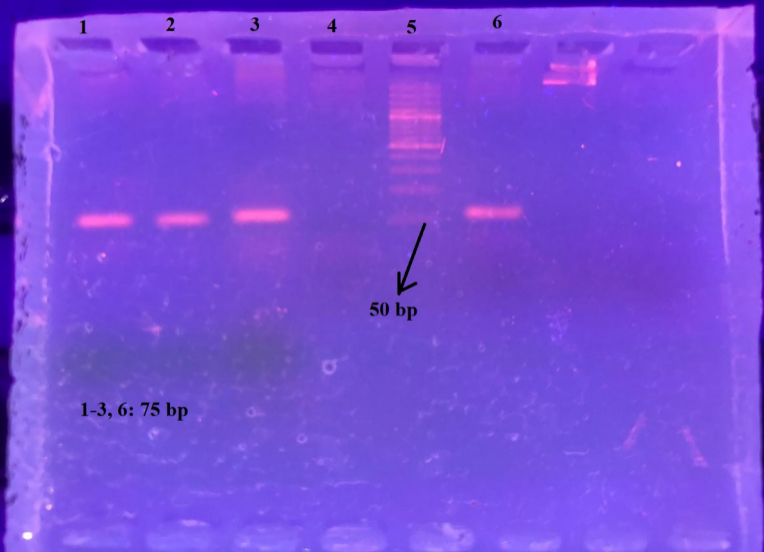

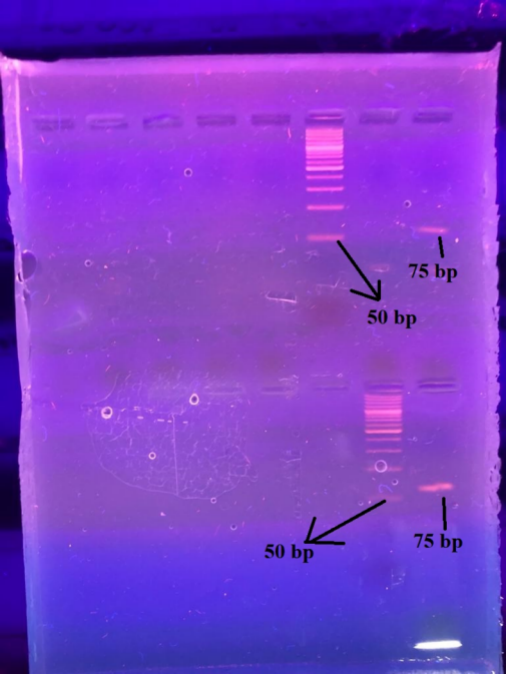

75 bp  
50 bp

50 bp  
75 bp

**i k l m**

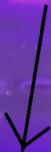

**50 bp**

**i-m: 75 bp**

Supplement: S1 Raw image — (PDF) [file pone.0295124.s002.pdf]
